# Fibro-Myomatous Tumours of the Uterus Producing Bladder Symptoms

**Published:** 1894-09-01

**Authors:** James Oliver

**Affiliations:** Physician to the Hospital for Women, London


### Medical Progress and Hospital Clinics.

### FIBRO-MYOMATOUS TUMOURS OF THE UTERUS PRODUCING BLADDER SYMPTOMS.

By James Oliver, M.D., F.R.S.Edin., Physician to the Hospital for Women, London.

**Case 1.—Fibro-Myoma of Uterus.**—Difficulty in micturition after a night's rest.

C. M., æt. 35 and single. Catamenia began at the age of 15, recurring regularly every three weeks for four days, and never profuse.

The patient complains of difficulty in micturition on rising in the morning, but there is no pain. The difficulty has existed ever since the patient had rheumatic fever two years ago.

*Physical Signs.*—The lower abdomen, especially on the right side, is rather prominent. It is occupied by an irregularly nodulated swelling which extends from the pubes to a spot midway between the pubes and the umbilicus. The swelling on the right side is composed of two portions, an anterior, which is a flaccid sac containing fluid, and a posterior, which is solid. At this stage the catheter was passed, and by this means the flaccid sac on the right side, which contained urine, was emptied, and underlying it were felt several small, hard projecting nodules.

The cervix uteri, which is short, is located towards the right side of the pelvis, and the vaginal roof generally is occupied by a hard, irregular mass, which is continuous with the abdominal swelling. The pelvic portion of the tumour is very fixed, especially on the right side.

*Remarks.*—It is, of course, impossible in this case to say how long the tumour may have been in existence before it was detected by me, but it is more than probable that it had already incorporated a considerable portion of the bladder with its structure when the patient had rheumatic fever—two years before coming under my observation. From the physical signs it was evident that the myomatous tumours in their growth had gradually attenuated that portion of the bladder which, under ordinary circumstances, is loosely connected with the pad of cellular tissue which joins the bladder to the lower third of the uterus. In this manner the expansive power of the bladder had become impaired, partly by attenuation and partly by fixation, but no difficulty in passing water was experienced until the general state of the patient was disturbed by an attack of rheumatic fever. Even then it would appear that, so long as undue distension was avoided, the bladder was capable of fulfilling its function without attracting attention. In the morning, however, after a night's rest, the already enfeebled organ was so disadvantageously circumstanced that micturition was always more or less difficult at this time of the day.

**Case 2.—Fibro-Myoma of Uterus** producing retention of urine after the menopause (this patient was sent to me by Dr. Greet, of Vernon Square).

Sarah H., æt. 55, and married 18 years; has had one child. She ceased menstruating four years ago.

In May and December, 1892—three and three and a-half years respectively after the menopause—patient, without ever having previously experienced any trouble with the bladder, complained of retention of urine. On each of these occasions, however, she was eventually enabled to pass water without the use of the catheter. In April, 1893, she again consulted Dr. Greet in consequence of an inability to pass water, but on this occasion the former remedies proving inefficacious the bladder had to be emptied by the catheter, and for seven days before coming under my observation the water had thus been drawn off daily.

*Physical Signs.*—The abdomen is occupied on the right side by a small rounded swelling, which rises out of the pelvis and reaches to a point midway between the pubes and the umbilicus. The cervix, which is large, is located towards the right side of the pelvis, and is pressed against the pubes. Behind the cervix is felt a hard globular swelling which has pushed down the vaginal roof. This swelling is continuous with the abdominal tumour and with the uterus.

*Remarks.*—Fibro-myomatous tumours of the uterus may exist for years without producing any special disturbance, and without even the patient herself being aware of the existence of any abnormal growth. In the case Sarah H——, it is probable that the tumour had begun to develop soon after the child was born, and this event occurred ten years before the menopause. After the cessation of menstruation the majority of these tumours of the uterus not only cease to grow but tend to diminish in size, and consequently it is unusual to find symptoms arising from their presence during this period of life. Occasionally, however, it happens that fibro-myomatous tumours degenerate after the functional activity of the uterus declines, or hæmorrhagic cysts may even then develop in their substance, and symptoms may of course be engendered by such changes. In the case now under consideration the physical examination failed to detect any evidence of retrograde change in the growth itself, but showed that the tone of the bladder, which had gradually become impaired, as in an ordinary case of prolapse of the uterus, was now unable to overcome the resistance offered by the tumour, which had sunk considerably in the pelvis.

**Case 3.—Fibro-Myoma of Uterus.**—Incontinence of urine just before menstruation, difficulty in passing at other times.

E. J——, æt. 42, and married ten years, has never been pregnant. She began to menstruate at the age of twelve, and the discharge, which has always been large in amount, has during the last six months become greatly augmented. For eighteen months patient has complained of an inability to hold the water immediately before each menstrual period, and of difficulty in passing it at other times.

*Physical Signs.*—The abdomen is occupied by a central and irregularly globular swelling, which rises from the pelvis and reaches to 1½ inches above the umbilicus. It is not hard, though solid. The vaginal roof anteriorly is drawn up above the level of the pubic bone. The cervix, which is flush with the vaginal roof, is located towards the left side of the pelvis, and about half an inch below it is a deep crescentic fold, which is formed by the posterior vaginal wall. The floor of Douglas's pouch, which is occupied by a swelling, has been pushed down to within 1½ inches of the orifice of the vagina.

*Remarks.*—The difficulty in passing water in this case was due to the incorporation of the bladder with the uterine tumour, as in Case 1, but the periodic variation in the state of the uterus induced either an irritable condition of the bladder or annulled by inhibition its sphincter, so that at or about the time of menstruation the patient was unable to exercise any control whatever over this viscus.

**Case 4.—Fibro-Myoma of Uterus,** causing retention of urine for three months.

E. D——, æt. 44, and married 10 years, has never been pregnant. There is nothing special to note about the menstrual history. She is quite regular. For two or three years patient has complained of swelling of the abdomen, especially on the left side. For 10 or 11 months she has experienced a "bearing down" sensation in the front and back passages. Fifteen months ago patient remarked that she had difficulty in passing water. Twelve months ago there was complete retention of urine for three months, so that the water had to be drawn off by the catheter every third day. For nine months again she has complained of an inability to pass water except when recumbent.

*Physical Signs.*—The abdomen is occupied by a small irregular and solid tumour which rises out of the pelvis and extends to three inches above the pubes. The cervix is fairly central, and in front of it is felt a large nodule which pushes down the anterior vaginal roof below the level of the cervix itself. The whole tumour is the enlarged uterus.

*Remarks.*—The retention of urine in this case was largely due to deficient tone in the pelvic tissues, and this tenet is confirmed by the fact that the patient was enabled to pass water fairly comfortably when she assumed the recumbent position.

